# The RNA Helicase DDX6 Associates with RIG-I to Augment Induction of Antiviral Signaling

**DOI:** 10.3390/ijms19071877

**Published:** 2018-06-26

**Authors:** Rocío Daviña Núñez, Matthias Budt, Sandra Saenger, Katharina Paki, Ulrike Arnold, Anne Sadewasser, Thorsten Wolff

**Affiliations:** Robert Koch-Institut, FG17—Division of Influenza Viruses and other Respiratory Viruses, 13353 Berlin, Germany; rociodavinha@outlook.com (R.D.N.); budtm@rki.de (M.B.); saengers@rki.de (S.S.); katharina.paki@boehringer-ingelheim.com (K.P.); arnoldu@rki.de (U.A.); anne.sadewasser@secarna.com (A.S.)

**Keywords:** DDX6, RIG-I, influenza, interferon

## Abstract

Virus infections induce sensitive antiviral responses within the host cell. The RNA helicase retinoic acid-inducible gene I (RIG-I) is a key sensor of influenza virus RNA that induces the expression of antiviral type I interferons. Recent evidence suggests a complex pattern of RIG-I regulation involving multiple interactions and cellular sites. In an approach employing affinity purification and quantitative mass spectrometry, we identified proteins with increased binding to RIG-I in response to influenza B virus infection. Among them was the RIG-I related RNA helicase DEAD box helicase 6 (DDX6), a known component of cytoplasmic mRNA-ribonucleoprotein (mRNP) granules like P-bodies and stress granules (SGs). RIG-I and DDX6 both localized to the cytosol and were detected in virus-induced SGs. Coimmunoprecipitation assays detected a basal level of complexes harboring RIG-I and DDX6 that increased after infection. Functionally, DDX6 augmented RIG-I mediated induction of interferon (IFN)-β expression. Notably, DDX6 was found to bind viral RNA capable to stimulate RIG-I. These findings imply a novel function for DDX6 as an RNA co-sensor and signaling enhancer for RIG-I.

## 1. Introduction

Influenza viruses (IVs) cause annual epidemics and occasional pandemics in the human population with severe impact on global health. While influenza A viruses (IAVs) occur in different subtypes and infect a range of other species, the monophyletic influenza B viruses are largely confined to humans. All IVs contain a segmented RNA genome of negative polarity. The viral RNAs are sensed by the cytoplasmic RIG-I sensor that initiates a signaling pathway leading to production of type I interferons (IFNs) and proinflammatory cytokines [1,2,3,4]. Type I IFNs activate the expression of several hundred gene products, many of which can confer an antiviral state to the cell [5,6]. The family of RIG-I-like receptors includes RIG-I, MDA5, and LGP2, all of which share a common DExD/H-box RNA-dependent helicase domain and a C-terminal domain (CTD) [7]. In addition, RIG-I and MDA5 have two N-terminal caspase recruitment domains (CARDs) which are the responsible signaling mediators. RIG-I senses short double-stranded RNA (dsRNA) with 5′-di- or triphosphate ends (5′-PP, or 5′-PPP, respectively) from different RNA viruses such as vesicular stomatitis virus, hepatitis C virus (HCV), as well as IV in many cell types [8,9,10,11,12,13]. Upon ligand recognition, RIG-I undergoes a conformational change from its autorepressed form into an active state by unmasking the CARD domains [7]. Oligomerization of RIG-I on RNA ligands drives the formation of helical CARD domain filaments which recruits and organizes the downstream adapter MAVS (also known as IPS-1, VISA, or Cardif) into similar filamentous aggregates [14]. This mediates activation of the transcription factors IRF3 and IRF7 through the phosphorylation by Iκ-B kinase family members TBK1 and IKKε. IRF3/7 translocates to the nucleus and activates the transcription of type I IFN [15]. A key mechanism for the regulation of RIG-I activation involves its activation by covalent K63 chain ubiquitination mediated by the E3 ligases Riplet, TRIM25, Ube2D3, and MEX3C. Retinoic acid-inducible gene I is negatively controlled through phosphorylation by PKCs, DAPKI, and CKII [16,17,18,19,20,21,22,23,24]. Also several other interacting proteins, like PACT, ZAPS, and OASL have been shown to regulate RIG-I activation [25,26,27,28,29], but additional regulators may exist. In addition to the production of type I IFN and proinflammatory cytokines, cells have evolved other RNA-triggered defense mechanisms against viral infections. For instance, protein kinase R (PKR) is activated following binding of viral RNA and can induce translation arrest by phosphorylation of the eukaryotic translation initiation factor 2 alpha (eIF2α) [30]. Stalled translation machinery is then accumulated in stress granules (SGs), which are dynamic cytosolic aggregates that contain translationally arrested mRNAs, 40S ribosomes, and various RNA-binding proteins like TIA-1, TIAR, and G3BP1 [31,32]. Importantly, it has been recently suggested, that SGs serve as assembly platforms for the shaping of an antiviral host response and their presence correlates with reduced virus replication [33,34]. Thus, antiviral SGs (avSG) triggered by infection with IV, hepatitis C virus or coronaviruses also contain many antiviral proteins like RIG-I, MAVS, or PKR, indicating a crosstalk between these pathways occurring here. In addition, virus ribonucleoprotein complexes are present in SGs, yet how this contributes to the host antiviral response is not well established [35,36].

Viruses employ multiple strategies to evade these coordinated host defenses. Influenza viruses express the nonstructural protein 1 (NS1), a multifunctional protein that antagonizes the cellular defense response by diverse mechanisms. Thus, NS1 inhibits RIG-I dependent activation of type I *IFN* genes, in case of IAV by targeting TRIM25-mediated ubiquitination of RIG-I [18,37]. Furthermore, NS1 also prevents the activation of PKR [18,38,39]. By this means, IV is able to inhibit PKR-dependent translational arrest and avSG formation.

In this study, using affinity purification and mass spectrometry, we sought to identify RIG-I interaction partners in IV-infected cells. We report that DDX6, a member of the DExD/H-box RNA helicases family and a SG and P-body component, associates with RIG-I and augments RIG-I-dependent expression of type I IFN. DDX6 colocalized with RIG-I in SGs, however the function of DDX6 in RIG-I’s signaling pathway was independent of SG formation. DDX6 overexpression increased IFN-β production in a dose-dependent manner. Consistently, knockdown of DDX6 reduced IFN-β production. Finally, DDX6 bound IV RNA with the ability to stimulate RIG-I. Taken together, these results show that DDX6 is a novel RIG-I regulator that plays a positive role in RIG-I mediated production of type I IFN.

## 2. Results

### 2.1. DDX6 Interacts with RIG-I

To identify novel RIG-I regulators during IV infection, we employed affinity purification of RIG-I protein complexes in combination with quantitative mass spectrometry. By stable isotope labeling with amino acids in cell culture (SILAC), all proteins in a sample are marked with a distinct isotopic label. We performed triple-labeling SILAC experiments, in which three differentially labeled 293T cell cultures were transfected with a streptavidin-tagged RIG-I (STREP-RIG-I) construct and were either mock-infected, infected with influenza B wild type virus or with the NS1 mutant #4 strain. Due to the mutations R58A/K60A/K64A in its NS1 protein, the mutant virus is no longer able to inhibit PKR [40]. We included this mutant in our analysis because PKR has an important role for RIG-I activation [35], acting via the induction of SGs. Due to the different labels, peptides from each state generated in the MS work flow could be distinguished and quantified. Labeled cell lysates were combined in equal proportions, RIG-I complexes were purified over a STREP-tactin affinity column and eluted proteins were subjected to LC-MS/MS analysis. The experimental setup and the MS raw data are shown within Supplementary Table S1.

Protein hits were considered significant with a cutoff value of 2-fold overrepresentation in infected versus mock samples. In total, we identified binding of 180 proteins to RIG-I in wild type (wt) virus infected cells and 51 proteins in mutant #4 infected cells, respectively. Among those were known RIG-I interactors like 14-3-3ε and ubiquitin indicating the validity of our approach [41,42]. Still, we considered that candidate proteins need to be carefully evaluated to count as bona fide RIG-I ligands as several factors including the overexpression of the RIG-I bait or the highly dynamic association of RIG-I with partially insoluble complexes could have influenced the outcome of the experiments. Among the identified proteins we focused our further analysis on DDX6 since, like RIG-I, it belongs to the DExD/H box RNA helicases. This family comprises many proteins involved in mRNA metabolism, and several of its members in addition to the well-established RIG-I-like helicases RIG-I, MDA5, and LGP2 have been recently shown to be involved in antiviral immunity.

**Figure 1:**
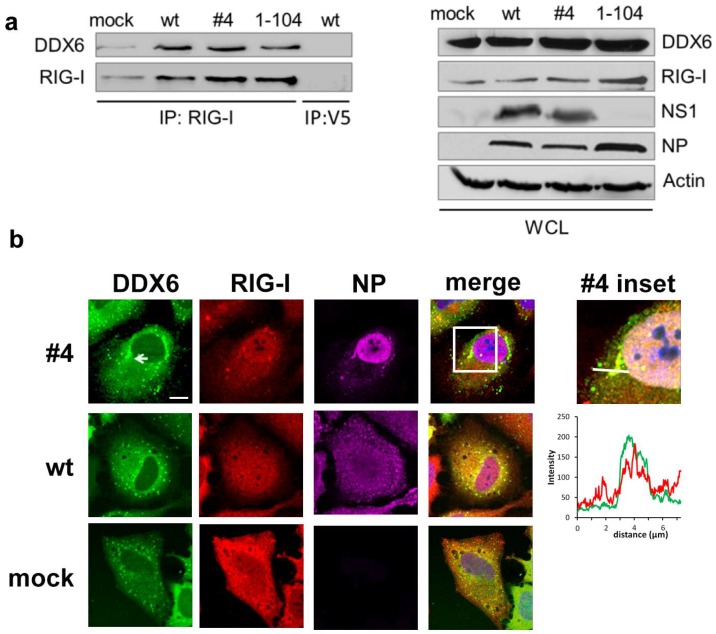
Physical interaction and colocalization of RIG-I and DDX6. (**a**) HeLa cells were mock treated, infected with wild type or the NS1 mutant #4 or 1–104 viruses. Sixteen hours after infection, RIG-I was immunoprecipitated with a RIG-I-specific antibody and anti-V5 antibody was used as a negative control. The precipitated proteins (IP, top) and input lysates (WCL, bottom) were analyzed by immunoblotting using the indicated antibodies. The truncated NS1 1–104 protein was not detectable by the antiserum used, but its expression has been characterized elsewhere [40] A representative experiment of *n* = 4 is shown. (**b**) HeLa cells transfected with Flag-RIG-I and GFP-DDX6 were mock treated or infected with either influenza B wild type or NS1 mutant #4 virus. Sixteen hours post infection, cells were fixed and stained for RIG-I (red channel) and DDX6 (green channel). Infection was verified by staining of influenza virus nucleoprotein (NP; magenta channel). Nuclei were visualized by DAPI staining. Images were taken by confocal microscopy. The square area of the inset is digitally magnified on the right hand side (inset). The intensity profiles of the green and red channel at the location of the bar in the inset are shown on the bottom. Scale bar, 10 μm.

First, we confirmed the interaction between RIG-I and DDX6 by coimmunoprecipitation (Co-IP) assays of the endogenous proteins RIG-I and DDX6 in normal and virus-infected cells. In this set of experiments we also included the NS1 1–104 mutant virus, in which NS1 amino acids 105–281 are truncated. This mutant strain is able to inhibit PKR but defective in limiting IFN-β production [40], enabling us to compare the role of DDX6 in cells with silent (uninfected or wt-virus infected) versus activated RIG-I (NS1 1–104 mutant virus). HeLa cells were mock-treated or infected with wild type, the #4 or NS1 1–104 mutant viruses, respectively, and RIG-I was immunoprecipitated from cell lysates with a specific antibody (Figure 1a). While DDX6 weakly coprecipitated with RIG-I in mock-treated cells, both the signal for RIG-I and associated DDX6 were increased in a similar manner in infected cells. The physical association of DDX6 with RIG-I was confirmed in a reverse Co-IP approach, in which RIG-I associated with immunoprecipitated DDX6 (Figure S1). These findings demonstrate the constitutive presence of RIG-I- and DDX6-containing complexes, whose abundance increases in infected cells.

### 2.2. DDX6 Colocalizes with RIG-I in Stress Granules

**Figure 2:**
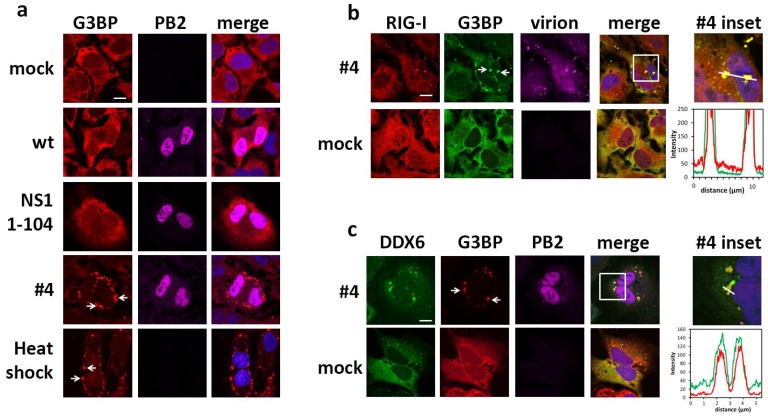
Mutant influenza virus infection induces stress granule association of RIG-I and DDX6 (**a**) HeLa cells were either mock treated, infected with influenza B wild type virus, NS1 mutant viruses 1–104 or #4, respectively, for 16 h, or were incubated at 46 °C for 40 min. (heat shock). Subsequently, cells were fixed and stained with G3BP and influenza virus PB2 specific antibodies. Nuclei were visualized by staining with DAPI. Arrows mark G3BP-positive granules (**b**,**c**), HeLa cells transfected with Flag-RIG-I and GFP-DDX6 were mock treated or infected with NS1 mutant #4 virus. Sixteen hours post infection, cells were fixed and stained for RIG-I, DDX6 and with antibody directed against influenza B virions (**b**) or PB2 (**c**). Images were analyzed by confocal microscopy. Arrows mark G3BP-positive granules in which also RIG-I (**panel b**) or DDX6 (**panel c**) were detected. The square area of the inset is digitally magnified on the right hand side (inset). The intensity profiles of the green and red channels are shown on the bottom. Scale bar, 10 μm.

Next, we addressed the intracellular localization of RIG-I and DDX6 by confocal microscopy. HeLa cells transfected with Flag-RIG-I and GFP-DDX6 were mock treated or infected either with the wild type or mutant #4 virus unable to silence PKR activation. In non-infected cells, DDX6 presented a diffuse staining throughout the cytosol similar to RIG-I and was, in addition, detected in a punctate pattern (Figure 1b). Cells infected with the wild type virus presented a similar staining as the non-infected cells. Interestingly, in mutant #4 virus infected cells, RIG-I and DDX6 colocalized in larger dot-like structures (Figure 1b) in addition to a diffuse staining throughout the cytosol. Since mutant #4 virus is unable to inhibit PKR, we hypothesized that the dot-like cytosolic structures corresponded to SGs. In fact, only cells subjected to heat shock [43] or infected with the mutant #4 virus harbored large cytosolic granules containing the SG marker protein G3BP (Figure 2a), indicating that mutant #4 virus induces SGs. While only 3% (5/152) of mock cells and 4% (5/117) of wt-infected cells displayed a granular G3BP pattern, 59% (65/110) of mutant #4-infected cells were SG positive. To verify the expectation that RIG-I and DDX6 also accumulate in SGs, we infected transfected HeLa cells with the mutant #4 virus. The infected cells presented SGs in which G3BP colocalized with RIG-I (Figure 2b) as well as with DDX6 (Figure 2c). These results show that RIG-I and DDX6 presented a diffuse distribution throughout the cytosol in wild type virus infected cells and colocalized in SGs, when cells were infected with the mutant influenza B virus. Based on the phenotype of the NS1 mutant virus, these findings also establish that the NS1 protein of influenza B virus has a conserved capacity to control SG formation, although it shares less than 25% sequence identity with IAV orthologues [33,44].

### 2.3. DDX6 Enhances RIG-I Mediated IFN-β Gene Expression

**Figure 3:**
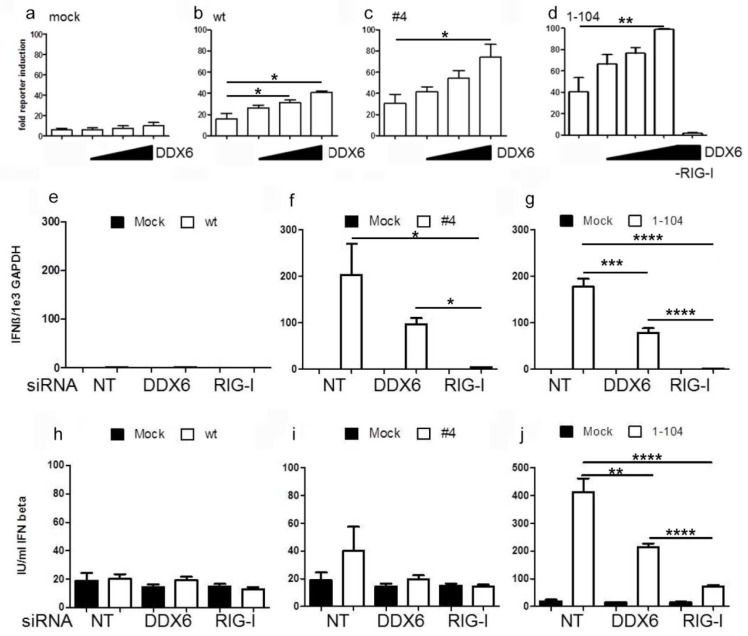
DDX6 promotes RIG-I mediated IFN-β induction. 293T cells co-transfected with Flag-RIG-I, GFP-DDX6 in increasing amounts (0, 50, 100, 150 ng), the p125-Luc pIFN-β firefly luciferase reporter and a Renilla control luciferase plasmid were mock treated or infected with the indicated virus for 16 h. IFN-β promoter activation was determined by measurement of luciferase activity. Ratios of firefly and corresponding Renilla luciferase activities were calculated and expressed relative to maximal activation, which was set as 100% (**a**–**d**). (**e**–**j**) SiRNA-mediated knockdown of endogenous DDX6 reduces RIG-I-mediated IFN-β induction. A549 cells were transfected with the indicated siRNA for 48 h and infected with the indicated virus. At 16 h post infection (hpi), cellular IFN-β mRNA levels were determined by qRT-PCR and normalized to GAPDH levels (**e**–**g**) or the concentration of IFN-β in the corresponding cell supernatants was determined by ELISA (**h**–**j**). Values are mean ± SD of at least three independent experiments (* *p* ˂ 0.05, ** *p* ˂ 0.01, *** *p* ˂ 0.001, **** *p* ˂ 0.0001).

Having established an interaction of DDX6 and RIG-I, we next addressed a putative function of DDX6 in RIG-I-mediated signaling. Initially, we analyzed the influence of DDX6 on RIG-I-dependent IFN-β induction in response to IV infection in an established reporter assay [4]. We found that DDX6 expression significantly upregulated IFN-β promoter activation by the wild type and the NS1 mutant viruses #4 and 1–104 in a dose-dependent manner (Figure 3a–d). This effect was completely dependent on RIG-I, as expression of DDX6 alone did not enhance reporter activation (Figure 3d, last column). As expected, levels of IFN-β were on average higher for the NS1 #4 and 1–104 virus infected cells, compared to wild type infection, due to the inhibitory activity of the NS1 wild type protein [40].

We confirmed the regulatory role of DDX6 in RIG-I-dependent IFN-β production in a complementary loss-of-function approach. Immunoblotting analysis showed that specific siRNAs efficiently reduced expression of DDX6 or RIG-I in human A549 lung epithelial cells (Figure S2). Cells infected with the wild type virus did not upregulate IFN-β on the transcript and cytokine levels, as expected [40] (Figure 3e,h). RIG-I knockdown reduced the production of IFN-β mRNA in response to NS1 mutant virus infection by up to 90%, whereas knockdown of DDX6 decreased IFN-β mRNA production by about 50–60% (Figure 3f,g). Measurement of IFN-β concentrations in the supernatant of infected DDX6 knockdown cells by ELISA revealed corresponding reductions in cytokine secretion (Figure 3j). Taken together, these findings corroborate a novel role for DDX6 as an enhancer of RIG-I signaling. Moreover, they are suggestive for a SG-independent mechanism of DDX6 since its influence on RIG-I-mediated IFN-β induction was also observed in wild type and NS1 1–104 virus-infected cells that both did not show enhanced SG formation.

### 2.4. RIG-I Binds DDX6 via Its CARD Domains

**Figure 4:**
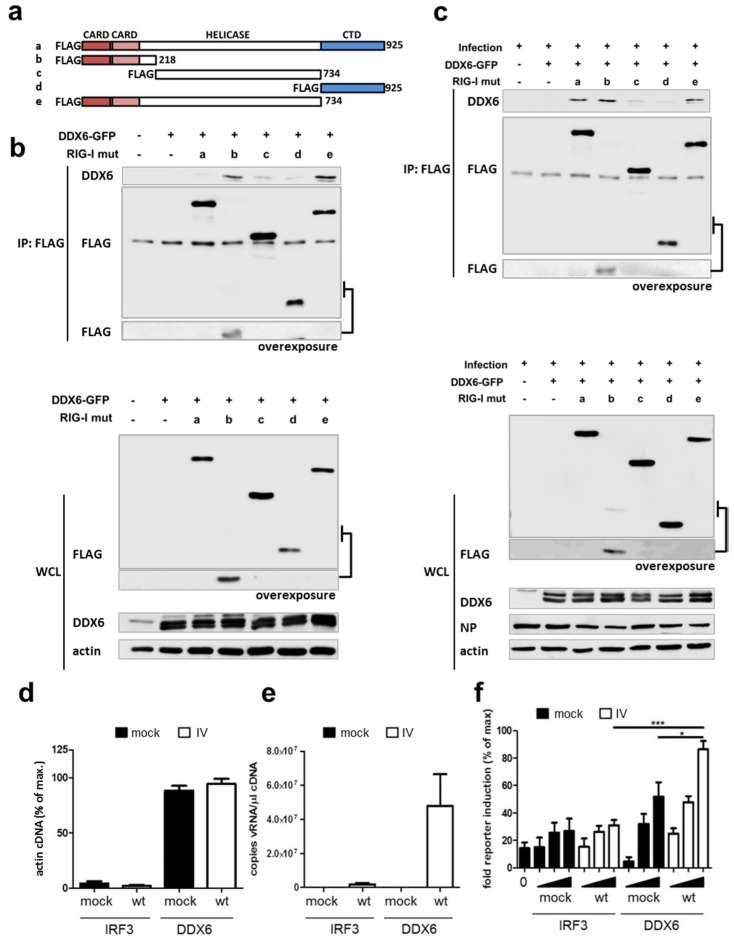
Analysis of RIG-I interactive domains and binding of viral RNA by DDX6. (**a**) Schematic diagram of RIG-I wild type and mutant proteins indicating the two CARD domains, residues 1–87 and 92–172, respectively), a central helicase domain (residues 251–776) and a C-terminal repressor domain (CTD, residues 735–925). (**b**,**c**) 293T cells co-transfected with GFP-DDX6 and full-length or mutant Flag-RIG-I were either mock-infected (**b**) or infected with Influenza NS1 1–104 virus (**c**). At 16 h post infection, RIG-I was immunoprecipitated from cell lysates with anti-flag agarose beads. The immunoprecipitated proteins (IP, top) and the input (WCL, bottom) were detected by immunoblotting analysis using the indicated antibodies. For the indicated areas of the FLAG blots, additional longer exposures are shown to verify the presence of RIG-I CARD only mutant b (**b**,**c**). (**d**,**e**) 293T cells transfected with GFP-DDX6 or GFP-IRF3 were mock infected or infected with influenza B wild type virus (IV). Cell extracts were prepared at 16 h post infection and the expressed DDX6 or IRF3 proteins were selected by GFP-trap, followed by purification of the associated RNA. Subsequently, copy numbers of actin mRNA (**d**) or influenza B virus RNA derived from the viral M segment (**e**) were determined by qRT-PCR. (**f**) RNA eluted from GFP-DDX6 or GFP-IRF 3 precipitates as detailed in panel (**d**) was re-transfected in increasing amounts (10, 30, 100 ng) into 293T cells together with Flag-RIG-I, p125-Luc and a control Renilla luciferase. Cells were harvested 16 h post transfection and the RIG-I-mediated IFN-β activation was determined by reporter assay. Values are mean ± SD of at least three independent experiments (* *p* ˂ 0.05, *** *p* ˂ 0.001).

To understand the molecular organization of the RIG-I-DDX6 complexes we determined the domains of RIG-I involved in the interaction with DDX6 by coimmunoprecipitation using a set of RIG-I deletion mutants, lacking the CARD-, helicase- or C-terminal domains (Figure 4a). In non-infected cells, only the isolated CARD domains (mutant b) and the ΔCTD construct (mutant e) were able to efficiently pulldown DDX6, whereas the full-length RIG-I protein showed only very weak binding (Figure 4b). In contrast, in mutant NS1 1–104-infected cells, in addition to the CARD and ΔCTD domains, also the full-length RIG-I protein was able to associate with DDX6 (Figure 4c). The lack of the regulatory CTD in mutants b and e is expected to expose the CARD domains and allow binding to DDX6 in the absence of infection [7]. In contrast, for the wt RIG-I protein (construct a), an infection with a virus defective in RIG-I inhibition is required to induce release of the CARD domains from its intramolecular sequestration. Thus, in this assay system, wt RIG-I only binds DDX6 in cells infected with NS1 1–104 virus. In conclusion, these findings indicate that the binding of DDX6 and RIG-I occurs primarily through the RIG-I CARD domains.

### 2.5. DDX6 Binds Viral RNA That Activates RIG-I

DDX6 associates with several types of RNA in the cell, including mRNA, miRNAs and also HCV RNA [45,46,47]. In an attempt to gain a better understanding of the mechanism underlying DDX6-mediated enhancement of RIG-I activation, we tested whether DDX6 was also able to bind IV RNA. To this end, cells transfected with GFP-tagged DDX6 were mock-treated or infected with wild type virus. GFP-tagged IRF3 was used as a negative control, since IRF3 is not known to bind RNA. Proteins were harvested from cell lysates by GFP affinity purification and RNA was isolated from these complexes by proteinase K digestion and column purification. The eluted RNAs were first examined by qRT-PCR showing that DDX6 bound comparable amounts of cellular actin mRNA irrespective of infection indicating that similar RNA quantities were present in the different settings (Figure 4d). Interestingly, we found that DDX6 also captured substantial amounts of viral RNA from virus infected cells, while IRF3 did not (Figure 4e). We then hypothesized that the DDX6-associated viral RNA might activate RIG-I. To test this, RNA eluted from DDX6 or IRF3 as shown in Figure 4d and e, was re-transfected into 293T cells and evaluated for RIG-I-dependent IFN-β promoter induction. Significantly, DDX6-bound RNA from infected cells specifically stimulated RIG-I-mediated IFN-β promoter activation in a dose-dependent manner (Figure 4f), demonstrating that DDX6 binds viral RNA that has the capacity to stimulate RIG-I.

## 3. Discussion

The current study aimed at a better understanding of the regulation of RIG-I activation, a central player in the cellular innate response against IV. Here, we identified the RNA helicase DDX6 as a novel interaction partner of RIG-I. The association of RIG-I with SGs and their role in the induction of an antiviral response has been established [35,48]. DDX6 is a well-known component of SGs, but apart from that has a pleiotropic role in the metabolism, distribution and storage of RNA in the cell [45,49]. Consistently, our coimmunoprecipitation experiments identified an interaction between RIG-I and DDX6 also in the absence of SGs. Further, our data argue against an RNA-bridged interaction of RIG-I and DDX6, since DDX6 directly binds to the RIG-I CARD domains. This binding pattern suggests a preference of DDX6 to bind to activated RIG-I, in which the CARD domains are released from intramolecular sequestration [50] and become available for protein-protein interactions. Also, several of DDX6′ established properties could enable it particularly well to serve as an auxiliary factor for RIG-I. DDX6 is constitutively expressed in cells at high concentrations (estimated 2 × 10^6^ DDX6 proteins per HeLa cell) and it binds RNA sequence-independently with nanomolar affinity [51]. DDX6 represses mRNA translation by binding mRNAs and storing them in P-bodies. Since DDX6 has the capacity to unwind secondary structures in RNA [51,52], it is tempting to speculate that it can collect the viral RNA and present it in suitable topology to RIG-I to induce its activation. This would also match to the recently reported activity of RIG-I not only as a sensor but also as an active disruptor of viral ribonucleoprotein complexes [53,54,55]. In the future, it will be important to untangle the interplay of DDX6 and mRNP granules in the initiation of antiviral responses. Of note, a similar role was identified for the P-body component LSm14A, which underscores the importance of mRNP granules as initiator sites of the host antiviral response [56]. Also in line with these findings, influenza A virus disrupts P-bodies during the course of infection [34], illustrating the complex interplay of cellular responses and viral countermeasures. DDX6 was recently shown to limit expression of interferon-stimulated genes (ISG) via an MDA5-dependent mechanism [57]. Thus, DDX6 might work as a buffer, supporting IFN induction via RIG-I and at the same time limiting detrimental effects of aberrant ISG expression.

Apart from DDX6, also other members of the DExD/H helicase family play important roles in antiviral responses, linking mRNP function and RIG-I signaling. Thus, DHX36 facilitates RIG-I signaling by inducing SG formation [58]. Similarly, DDX60 was shown to be important for recognition of VSV, Poliovirus, HSV-1 and SeV [59], and DDX3 and DDX21 control IAV replication, at least the former via regulation of SG formation [60,61,62]. Other members of this family exert negative regulatory roles on the IFN circuit [28]. We conclude that DDX6 belongs to a growing group of RNA helicases with versatile roles in the recognition of foreign RNA and the shaping of an innate antiviral host response.

## 4. Materials and Methods

### 4.1. Cells and Viruses

HeLa, A549, and 293T cells were maintained in Dulbecco’s modified Eagle’s medium (DMEM; MP Biomedicals, Eschwege, Germany) supplemented with 10% fetal bovine serum (FBS, Biochrom, Berlin, Germany) and 2 mM L-glutamine (Roth, Karlsruhe, Germany) at 37 °C in 5% CO_2_ atmosphere. The recombinant influenza B/Lee/40 and the isogenic mutant viruses NS1 1–104 and NS1 #4 have been described elsewhere and were propagated in embryonated chicken eggs [40]. For virus infection, cells were washed with PBS and infected with the virus diluted in PBS supplemented with 0.1% CaCl_2_, 0.1% MgCl_2_, and 0.2% bovine albumin (BA). After adsorption for 45 min at room temperature, cells were incubated in DMEM containing 0.2% BA at 33 °C in a 5% CO_2_ atmosphere. For heat shock treatment, cells were incubated for 1 h at 44 °C.

### 4.2. Plasmids

IFN-β promoter luciferase reporter plasmids, expression plasmids for Flag-tagged RIG-I were previously described [40]. GFP-tagged DDX6 was purchased from Addgene (plasmid 25033; Cambridge, MA, USA). The STREP-RIG-I expression plasmid was constructed by standard molecular biology techniques using the pESG-IBA-105 vector (iba GmbH, Goettingen, Germany).

### 4.3. RNA Interference

Transfections of siRNA were performed using Lipofectamine RNAiMAX (Invitrogen, Carlsbad, CA, USA) with 20 pmol of siRNA (Santa. Cruz Biotechnology, Heidelberg, Germany) for 0.2 × $10^{6}$ cells in 24 well plates according to the manufacturer’s instructions for 48 h. RIG-I-specific and control siRNA have been described elsewhere [4] and the siRNA 5′-AAGCAGAAACCCUAUGAGAtt-3′ (sense strand) was used to knock down DDX6 expression [63].

### 4.4. Immunoprecipitation and Antibodies

293T cells were transfected in 6-well plates with Lipofectamine 2000 (Invitrogen, Carlsbad, CA, USA) with plasmids encoding FLAG-tagged RIG-I and GFP-tagged DDX6 constructs. At 24 h after transfection, cells were lysed with lysis buffer (50 mM HEPES, 150 mM NaCl, 1 mM EDTA, 1% Igepal, 2 mM Na_3_VO_4_ supplemented with Protease Inhibitor Cocktail (Sigma-Aldrich, Taufkirchen, Germany), and proteins were immunoprecipitated with rabbit anti-FLAG (Sigma) antibody-coupled agarose beads. The beads were washed 4 times with lysis buffer and analyzed by SDS-PAGE and Western blotting with antibodies to Flag (mouse, Sigma-Aldrich), DDX6 (rabbit, Biomol, Hamburg, Germany) and Actin (mouse, Santa Cruz Biotechnology, Heidelberg, Germany). For the purification of DDX6-associated RNA, 293T cells in 10 cm dishes were transfected with DDX6-GFP or IRF3-GFP plasmids for 24 h and then infected with influenza B virus for 16 h (MOI = 3). After cell lysis, proteins were rotated with GFP-TRAP matrix at 4 °C for 2 h, followed by four washes of the beads with lysis buffer. RNA was isolated by proteinase K digestion and purification on RNeasy Mini spin columns (Qiagen, Hilden, Germany). For the coimmunoprecipitation of endogenous proteins, HeLa cells were plated in 10 cm dishes and infected with the indicated virus. 16 h after infection, cells were lysed and lysates were incubated with 10 µg of mouse monoclonal anti-RIG-I antibody (Enzo Life Sciences, Lörrach, Germany) at 4 °C for 2 h. The beads were washed 3 times and proteins were analyzed by immunoblotting with anti-RIG-I (Enzo Life Sciences), anti-DDX6 (Biomol), or anti-NP (Serotec, Düsseldorf, Germany) antibodies, respectively.

### 4.5. Confocal Microscopy

Cells grown on glass coverslips were fixed with 2.5% formaldehyde for 20 min, permeabilized with 0.2% Triton X-100 in PBS for 15 min and stained with monoclonal antibodies to Flag (mouse, Sigma) and/or G3BP (mouse, Becton Dickinson, Heidelberg, Germany) for 1 h at RT. AlexaFluor-conjugated secondary antibodies (Molecular Probes, Eugene, OR, USA) were used at 1:1000 dilution. Nuclei were counterstained with 4′,6-Diamidin-2phenylindol (DAPI). Cells were analyzed on a Zeiss LSM 780 laser scanning confocal microscope (Zeiss, Jena, Germany) using a 63× oil immersion objective with a numerical aperture of 1.4. Images were obtained with Zeiss Zen software (version ZEN 2012, Zeiss, Jena, Germany) and processed with Adobe Photoshop CS5 (Adobe Systems, San José, CA, USA).

### 4.6. Quantitative RT-PCR

Total RNA was extracted from cells with RNeasy kit (Qiagen, Hilden, Germany), reverse transcribed with oligo-dT18 using RevertAid reverse transcriptase (Thermo-Fisher Scientific, Waltham, MA, USA) and analyzed by Taqman qRT-PCR for IFN-β (primers 5′-CGCCGCATTGACCATCTA-3′ and 5′-GACATTAGCCAGGAGGTTCTCA-3′, probe YAK-5′-TCAGACAAGATTCATCTAGCACTGGCTGGCTGGA-3′), or GAPDH (primers 5′-GTTCGACAGTCAGCCGCATC-3′ and 5′-GGAATTTGCCATGGGTGGA-3′, probe FAM-5′-ACCAGGCGCCCAATACGACCAA-3′). DDX6- or IRF3-associated RNA was reverse transcribed with the influenza B virus M segment specific primer BMP-13 (5′-GAGACACAATTGCCTACCTGC-3′). The cDNA was analyzed by Taqman qRT-PCR with primers BMP-13 and BMP-102AN (5′-TTCCCACCGAACCAACAGTGTAAT-3′) and probe BM-72 (5′-CTGCTTTGCCTTCTC-3′) including a VIC fluorescent dye and a minor groove binder. For actin qPCR, oligo-dT18 was used for RT followed by PCR with primers 5′-AGCCTCGCCTTTGCCGA-3′ and 5′-CTGGTGCCTGGGGCG-3′ and probe FAM-5′-CCGCCGCCCGTCCACACCCGCC-3′. Standard curves were generated from PCR products cloned into pCRII-TOPO vector.

### 4.7. Statistical Analysis

All error bars represent standard deviations calculated from values obtained in at least 3 different experiments. Data were analyzed for statistical significance by the Mann–Whitney *U* test. A *p* value of less than 0.05 was considered significant.

### 4.8. IFN-β Luciferase Reporter Assay

293T cells seeded on 12-well plates were transfected with 100 ng of the IFN-β promoter plasmid p125-Luc, 10 ng of pTK-RL constitutively expressing Renilla luciferase together with a total of 500 ng of the indicated expression vectors or empty control vector as described [64]. 24 h later, cells were infected with the indicated virus. 16 hours post infection, cells were harvested and the luciferase activities were determined with the Dual-luciferase reporter assay system (Promega, Madison, WI, USA). Relative IFN-β promoter activation was calculated by normalizing Firefly to corresponding Renilla luciferase activities.

### 4.9. Enyzme-Linked Immunosorbent Assay (ELISA)

Culture supernatants were collected and subjected to ELISA with human IFN-β kit (FUJIREBIO Inc., Hannover, Germany) according to manufacturer’s instructions.

### 4.10. Affinity Purification of RIG-I Complexes

For SILAC experiments, 293T cells were differentially labelled using unlabeled l-arginine (R0) and l-lysine (K0) for light (R0K0), l-(13C6)-arginine (R6) and l-(2H4)-lysine (K4) for medium (R6K4) and l-(13C6, 15N4)-arginine (R10) and l-(13C6, 15N2)-lysine (K8) for heavy isotopic labeling (SILANTES, Munich, Germany). After full incorporation of the labelled amino acids during at least five passages, cells were transfected with STREP-RIG-I with calcium phosphate as follows: 1.5 mL of 2× HBS (20 mM HEPES, 140 mM NaCl, 50 mM KCl, 0.7 mM Na_2_HPO_4_) were mixed with 30 μg of STREP-RIG-I plasmid. 30 μL of 2.5 M of CaCl_2_ were added and the mixture was added dropwise into the medium. Cells were incubated for 24 h prior to infection. Amounts are referred to 15 cm dishes and 2 × 10^7^ cells. After infection, cells were lysed in lysis buffer and equal amounts of proteins from each lysate were mixed. RIG-I complexes were purified over a STREP-tactin column according to manufacturer’s instructions (iba Life Sciences, Göttingen, Germany).

### 4.11. Mass Spectrometric Analysis

Proteins eluted from the STREP-tactin column were concentrated by centrifugal evaporation and treated with 10 mM DDT and 50 mM iodoacetamide to reduce and alkylate cysteine residues. Proteins were then separated by SDS-PAGE and stained with Coomassie Blue. The gel lane was cut into equal 10 slices and each of them was subjected to in-gel digestion with trypsin. Peptides were separated by reverse-phase liquid chromatography on a nanoLC (Proxeon ThermoFisher, Darmstadt, Germany) coupled to the LTQ-Orbitrap XL (Thermo Fisher) over a stepwise gradient from 2% to 40% between buffer A (0.2% formic acid in water) and buffer B (0.2% formic acid in acetonitrile). The spray voltage was set at 1.8 kV and the capillary temperature at 200 °C. Data-dependent acquisition was performed on the LTQ-Orbitrap using Xcalibur 2.0 software (Thermo-Fisher Scientific, Waltham, MA, USA) in the positive ion mode. Full scan MS spectra (from *m*/*z* 300 to 1700) were acquired in the FT-Orbitrap with a resolution of 30,000. The five most intense peptide ions were sequentially isolated for fragmentation by collision induced dissociation (CID) with a collision energy of 35%.

### 4.12. Data Processing and Analysis

MS/MS data were processed using the SEQUEST algorithm in Proteome Discoverer 1.4.0.288. Protein search parameters included precursor mass tolerance of ±10 ppm, with a fragment mass tolerance of ±0.8 Da. Trypsin was set as the enzyme specificity with a maximum of two missed cleavage sites. Cysteine carbamidomethylation was set as a fixed modification and the four SILAC labels (K + 8.014199, K + 4.025107, R + 10.008269, R + 6.020129) were set as variable modifications. The false discovery rate (FDR) for all peptide and protein identifications was fixed at 5%. Searches were performed against the Homo Sapiens (NCBI Ref Seq) database (Available online: https://www.ncbi.nlm.nih.gov/refseq/). SILAC ratios were normalized according to the RIG-I levels in each state.

## Supplementary Materials

Supplementary materials can be found at http://www.mdpi.com/1422-0067/19/7/1877/s1.
