# Supplementary material for: The RNA Helicase DDX6 Associates with RIG-I to Augment Induction of Antiviral Signaling
Source: Int J Mol Sci. 2018 Jun 26;19(7):1877. doi: 10.3390/ijms19071877 (PMC6073104; doi:10.3390/ijms19071877)
Supplement: Supplementary file 1 [file ijms-19-01877-s001.zip › supplementary figures .pdf]

Figure S1

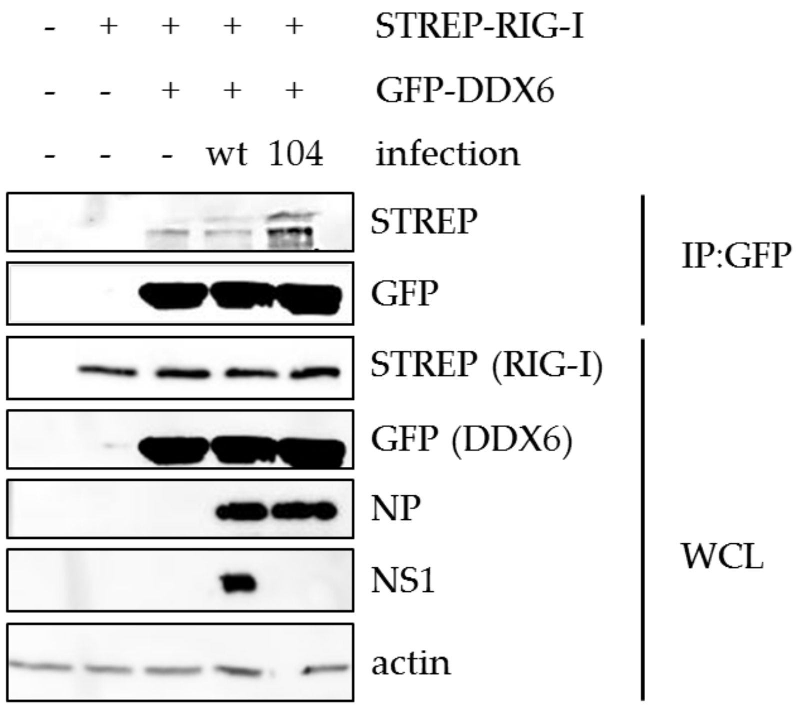

Figure S1. Physical interaction of DDX6 with RIG-I. 293T cells transfected with STREP-RIG-I and GFP-DDX6 were infected with influenza B wild type virus, NS1 mutant 1-104, or mock treated. 16 h after infection cells were lysed and DDX6 was immunoprecipitated. Proteins were analyzed by SDS-PAGE and immunoblot with the indicated antibodies.

Figure S2

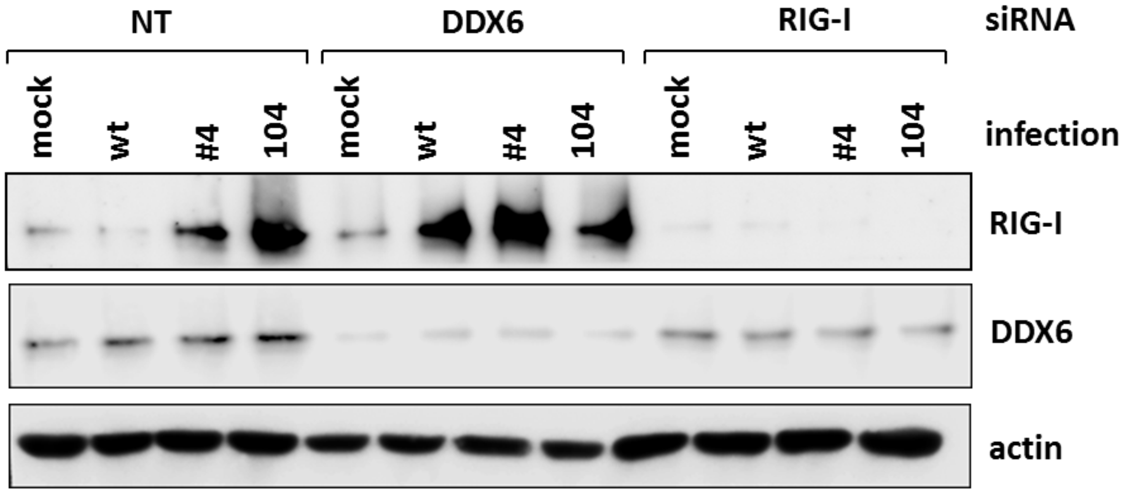

Figure S2. Si-RNA-mediated downregulation of RIG-I and DDX6 expression. A549 cells were transfected with siRNA directed against DDX6, RIG-I or non-target (NT) control, respectively. 48 h post transfection, cells were lysed and subjected to immunoblotting analysis with antibodies detecting DDX6, RIG-I or actin, respectively.
